# 3 Hand Burn Injuries and Occupational Impairment: Impact of Burn Injuries on Return-to-Work Outcomes

**DOI:** 10.1093/jbcr/irae036.003

**Published:** 2024-04-17

**Authors:** Barclay T Stewart, Bernadette Nedelec, Charles M Kopp, Nikhitha Thrikutam, Caitlin M Orton, Alyssa M Bamer, Jeffrey C Schneider, Kyra Solis-Beach, Lewis E Kazis, Haig A Yenikomshian, Karen J Kowalske

**Affiliations:** University of Washington, Seattle, WA; McGill University, Montreal, QC; Oregon Health Sciences University, Portland, Oregon; UW Medicine Regional Burn Center, Harborview Medical Center, Seattle, WA; University of Washington, Golden, Colorado; Spaulding Rehabilitation Hospital/Harvard Medical School, Boston, MA; UT Southwestern Medical Center, Dallas, Texas; Department of Health Law, Policy and Management, Boston University School of Public Health, Boston, MA; University of Southern California, Los Angeles, CA; University of Washington, Seattle, WA; McGill University, Montreal, QC; Oregon Health Sciences University, Portland, Oregon; UW Medicine Regional Burn Center, Harborview Medical Center, Seattle, WA; University of Washington, Golden, Colorado; Spaulding Rehabilitation Hospital/Harvard Medical School, Boston, MA; UT Southwestern Medical Center, Dallas, Texas; Department of Health Law, Policy and Management, Boston University School of Public Health, Boston, MA; University of Southern California, Los Angeles, CA; University of Washington, Seattle, WA; McGill University, Montreal, QC; Oregon Health Sciences University, Portland, Oregon; UW Medicine Regional Burn Center, Harborview Medical Center, Seattle, WA; University of Washington, Golden, Colorado; Spaulding Rehabilitation Hospital/Harvard Medical School, Boston, MA; UT Southwestern Medical Center, Dallas, Texas; Department of Health Law, Policy and Management, Boston University School of Public Health, Boston, MA; University of Southern California, Los Angeles, CA; University of Washington, Seattle, WA; McGill University, Montreal, QC; Oregon Health Sciences University, Portland, Oregon; UW Medicine Regional Burn Center, Harborview Medical Center, Seattle, WA; University of Washington, Golden, Colorado; Spaulding Rehabilitation Hospital/Harvard Medical School, Boston, MA; UT Southwestern Medical Center, Dallas, Texas; Department of Health Law, Policy and Management, Boston University School of Public Health, Boston, MA; University of Southern California, Los Angeles, CA; University of Washington, Seattle, WA; McGill University, Montreal, QC; Oregon Health Sciences University, Portland, Oregon; UW Medicine Regional Burn Center, Harborview Medical Center, Seattle, WA; University of Washington, Golden, Colorado; Spaulding Rehabilitation Hospital/Harvard Medical School, Boston, MA; UT Southwestern Medical Center, Dallas, Texas; Department of Health Law, Policy and Management, Boston University School of Public Health, Boston, MA; University of Southern California, Los Angeles, CA; University of Washington, Seattle, WA; McGill University, Montreal, QC; Oregon Health Sciences University, Portland, Oregon; UW Medicine Regional Burn Center, Harborview Medical Center, Seattle, WA; University of Washington, Golden, Colorado; Spaulding Rehabilitation Hospital/Harvard Medical School, Boston, MA; UT Southwestern Medical Center, Dallas, Texas; Department of Health Law, Policy and Management, Boston University School of Public Health, Boston, MA; University of Southern California, Los Angeles, CA; University of Washington, Seattle, WA; McGill University, Montreal, QC; Oregon Health Sciences University, Portland, Oregon; UW Medicine Regional Burn Center, Harborview Medical Center, Seattle, WA; University of Washington, Golden, Colorado; Spaulding Rehabilitation Hospital/Harvard Medical School, Boston, MA; UT Southwestern Medical Center, Dallas, Texas; Department of Health Law, Policy and Management, Boston University School of Public Health, Boston, MA; University of Southern California, Los Angeles, CA; University of Washington, Seattle, WA; McGill University, Montreal, QC; Oregon Health Sciences University, Portland, Oregon; UW Medicine Regional Burn Center, Harborview Medical Center, Seattle, WA; University of Washington, Golden, Colorado; Spaulding Rehabilitation Hospital/Harvard Medical School, Boston, MA; UT Southwestern Medical Center, Dallas, Texas; Department of Health Law, Policy and Management, Boston University School of Public Health, Boston, MA; University of Southern California, Los Angeles, CA; University of Washington, Seattle, WA; McGill University, Montreal, QC; Oregon Health Sciences University, Portland, Oregon; UW Medicine Regional Burn Center, Harborview Medical Center, Seattle, WA; University of Washington, Golden, Colorado; Spaulding Rehabilitation Hospital/Harvard Medical School, Boston, MA; UT Southwestern Medical Center, Dallas, Texas; Department of Health Law, Policy and Management, Boston University School of Public Health, Boston, MA; University of Southern California, Los Angeles, CA; University of Washington, Seattle, WA; McGill University, Montreal, QC; Oregon Health Sciences University, Portland, Oregon; UW Medicine Regional Burn Center, Harborview Medical Center, Seattle, WA; University of Washington, Golden, Colorado; Spaulding Rehabilitation Hospital/Harvard Medical School, Boston, MA; UT Southwestern Medical Center, Dallas, Texas; Department of Health Law, Policy and Management, Boston University School of Public Health, Boston, MA; University of Southern California, Los Angeles, CA; University of Washington, Seattle, WA; McGill University, Montreal, QC; Oregon Health Sciences University, Portland, Oregon; UW Medicine Regional Burn Center, Harborview Medical Center, Seattle, WA; University of Washington, Golden, Colorado; Spaulding Rehabilitation Hospital/Harvard Medical School, Boston, MA; UT Southwestern Medical Center, Dallas, Texas; Department of Health Law, Policy and Management, Boston University School of Public Health, Boston, MA; University of Southern California, Los Angeles, CA

## Abstract

**Introduction:**

Return to work (RTW) after burn injury is dependent on many variables, including type and location of burn injury, access to care, and pre-injury mental and physical health. Noting that prior studies were limited by small sample sizes, we aimed to use a large database to explore the associations between hand burn severity, functional hand outcomes, and RTW post-injury.

**Methods:**

Data from a multicenter prospective longitudinal study were analyzed. Adults with burn injuries were classified into 5 groups ranking in severity of hand injury: (0) no hand burns, (1) single hand burn no grafting, (2) bilateral hand burn no grafting, (3) single hand burn requiring grafting, (4) bilateral hand burn requiring unilateral graft, (5) bilateral hand burn requiring bilateral grafts. Grafting was used as a proxy for burn severity. Self-reported employment status, Patient-Reported Outcomes Measurement Information System (PROMIS) Upper Extremity (UE) scores, and reported request for work accommodations were collected at discharge, 6, 12, and 24 months post-injury. Descriptive statistics and analysis of variance (ANOVA) with post-hoc Tukey Test were completed to examine differences in outcomes by hand injury severity.

**Results:**

A total of 4,621 participants met inclusion criteria. Group 5, those with the most severe burns, had significantly longer mean RTW times than Groups 0-3 (p < 0.005). Group 5’s average RTW (242.6, Standard deviation (SD): 417.9) was greater, however not significantly, compared to group 4 (175.8 SD: 207.0). At 6 months, the mean PROMIS UE scores for grafted groups (Group 3, 40.6 SD: 11.4; Group 5, 35.4 SD: 12.8) were significantly worse than non-grafted groups (Group 1, 46.8; Group 2, 45.0; (p < 0.0001). At 12 months, Groups 5 (39.1 SD: 13.6) and 1 (48.7 SD: 8.6) remained significantly different. At 24 months, mean PROMIS UE scores were worse for grafted groups, though differences were no longer significant compared to non-grafted groups. At every time point, the majority of respondents did not request accommodations for their injuries from their employers (p-value 0.26, 0.66, and 0.86 for 6, 12, and 24 months respectively).

**Conclusions:**

Burn severity plays a significant role in both RTW and hand function for participants with hand burns. Additionally, the lack of correlation between burn severity and request for work accommodations hints at the baseline vulnerability of these populations. These findings suggest a need for systematic improvements in the way these patients are cared for and re-integrated into the workforce.

**Applicability of Research to Practice:**

These findings suggest a complex association between burn location, severity, patient functionality, and RTW. Furthermore, this study unveiled the disconnect between burn severity and request for work accommodations. Our data points to the need for further study into current methods of post-burn vocational rehabilitation.

